# GABAergic inhibition in the human visual cortex relates to eye dominance

**DOI:** 10.1038/s41598-021-95685-1

**Published:** 2021-08-23

**Authors:** I. Betina Ip, Uzay E. Emir, Claudia Lunghi, Andrew J. Parker, Holly Bridge

**Affiliations:** 1 Wellcome Centre for Integrative Neuroimaging, FMRIB, Nuffield Department of Clinical Neurosciences, University of Oxford, Oxford, OX3 9DU UK; 2 Department of Physiology, Anatomy and Genetics, University of Oxford, Oxford, OX1 3PT UK; 3 School of Health Sciences, Purdue University, West Lafayette, Indiana 47907 USA; 4 Laboratoire des systèmes perceptifs, Département d’études cognitives, École normale supérieure, PSL University, CNRS, 75005 Paris, France; 5 Institut für Biologie, Otto-Von-Guericke Universität, 39120 Magdeburg, Germany

**Keywords:** Striate cortex, Neuroscience, Sensory processing, Visual system

## Abstract

Binocular vision is created by fusing the separate inputs arriving from the left and right eyes. ‘Eye dominance’ provides a measure of the perceptual dominance of one eye over the other. Theoretical models suggest that eye dominance is related to reciprocal inhibition between monocular units in the primary visual cortex, the first location where the binocular input is combined. As the specific inhibitory interactions in the binocular visual system critically depend on the presence of visual input, we sought to test the role of inhibition by measuring the inhibitory neurotransmitter GABA during monocular visual stimulation of the dominant and the non-dominant eye. GABA levels were measured in a single volume of interest in the early visual cortex, including V1 from both hemispheres, using a combined functional magnetic resonance imaging and magnetic resonance spectroscopy (combined fMRI-MRS) sequence on a 7-Tesla MRI scanner. Individuals with stronger eye dominance had a greater difference in GABAergic inhibition between the eyes. This relationship was present only when the visual system was actively processing sensory input and was not present at rest. We provide the first evidence that imbalances in GABA levels during ongoing sensory processing are related to eye dominance in the human visual cortex. Our finding supports the view that intracortical inhibition underlies normal eye dominance.

## Introduction

Eye dominance in the healthy visual system is the preference for using one eye over the other in a visual task^1^. Extreme eye dominance is associated with amblyopia, a neurodevelopmental disorder that causes a chronic loss of normal monocular and binocular function with an incidence of 2–4% in the general population^2–4^. Input from the two eyes is combined for the first time in the primary visual cortex to serve binocular vision. Therefore, processing at this stage is thought to be decisive in eye preference. Understanding the relationship between eye dominance and the brain is thus an opportunity to investigate the neural mechanisms used by the cerebral cortex to serve perception.

Evidence for a neural mechanism of eye dominance comes from study of the abnormal binocular visual system. In adults, double vision arises when input from the two eyes does not correspond and this is associated with severe discomfort and headaches. In contrast, children who grow up with one deviating eye often develop strabismic amblyopia but perceive only a single image, not two. This is because their visual system has prevented double images from reaching perception by making the input from the deviating eye non-visible and relaying the image from the non-deviating eye. Known as binocular suppression, this type of chronic suppression could be ‘nature’s way out of trouble’^5^. The drawback of suppression is that it is related to a loss of binocular visual function^6^, most notably for stereopsis and binocular summation. Hence, visual cortex suppression has been named as one of the prime causes of the perceptual deficits observed in amblyopia^7^.

Severely imbalanced vision causes both anatomical and functional abnormalities in the primary visual cortex^8^, with functional abnormalities likely maintained by GABAergic inhibition^9^. The strongest evidence in support of this view has come from pharmacological studies in amblyopic animal models. In strabismic amblyopic cats, localized application of the GABA_A_ receptor antagonist bicuculline to V1 reduced binocular suppression^10^. Similarly, in amblyopic rats, pharmacologically decreasing GABAergic signalling in the visual cortex has been linked to a recovery of normal structure and visual function in the amblyopic eye^11,12^. This recovery was blocked by cortical delivery of diazepam, a GABA_A_ agonist^11^. These studies provide strong support for a role of GABAergic inhibition in maintaining extreme eye dominance in the adult brain.

Amblyopic suppression may be an extreme form of normal binocular interaction, revealed in a subtle form in normal participants using binocular rivalry^9^. Binocular rivalry is a widely used method to quantify sensory eye dominance in the normal-sighted population^1^. It induces continuous perceptual alternations between incongruent images presented separately to the left or right eye^13,14^. Theoretical models suggest that intracortical inhibition could explain the pattern of perceptual dynamics^15,16^. In recent years, support for this view has come from studies applying magnetic resonance spectroscopy (MRS) to measure GABAergic inhibition in the human brain in the absence of task specific stimulation (‘rest’). Greater levels of resting GABA levels in the early visual cortex (EVC) are related to stronger perceptual suppression^17^, and slower, less frequent perceptual switches^18,19^. Pharmacological manipulations of GABA have shown a causal relationship between GABA and binocular rivalry dynamics: increasing GABA reduced perceptual switches and increased percept durations^18^ and perceptual suppression^18,20^. In addition, increases in eye dominance duration during binocular rivalry induced by short-term monocular deprivation have been correlated with lower resting GABA levels in the primary visual cortex^21^. These results have highlighted the role of baseline GABAergic inhibition in shaping the competitive interactions in the visual brain, yet the hypothesis that imbalances during active stimulation of each eye are related to eye dominance awaits critical support.

To test the long-standing prediction that imbalances in cortical inhibition during monocular visual response are related to eye dominance^9^, we set out to measure GABA during functional stimulation. Specifically, we wanted to quantify the ‘interocular’ difference in GABA during monocular stimulation of either the stronger or the weaker eye. Conventionally, MRS experiments measure GABA levels during the resting state, when participants have their eyes closed, or simply watch a movie with both eyes open. In absence of a specific task, such measurements at rest are thought to reflect the overall inhibitory tone in the brain area under study^22^. In contrast, our study takes advantage of recent advances in ultra-high field functional MRS methodology, that allow measurement of metabolite levels during task performance^23–26^. For all participants, we determined eye dominance both using binocular rivalry and the magnitude of interocular inhibition, manifest in the difference in GABA levels during stimulation of the stronger and weaker eyes respectively. Our results provide the first evidence that eye dominance in the human visual cortex is related to neural mechanisms of active interocular inhibition.

## Results

### Binocular rivalry quantifies eye dominance

**Figure 1:**
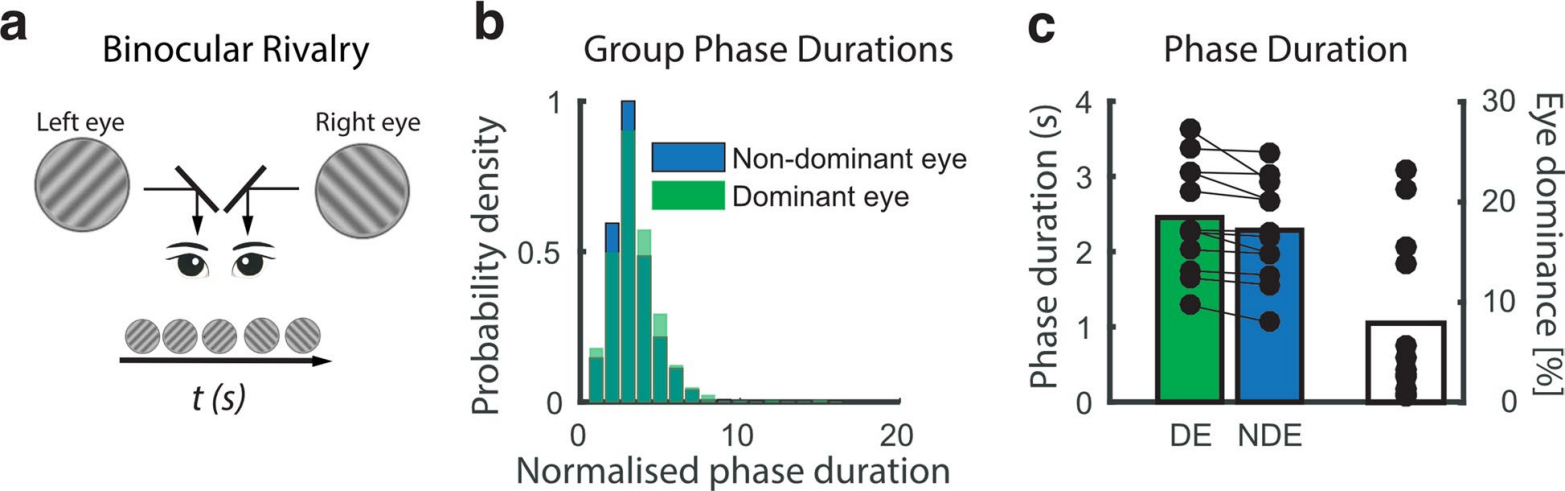
**(a)** Sensory eye dominance was measured in a binocular rivalry experiment outside of the scanner. Stimuli were presented on a mirror stereoscope and participants reported their subjective percept using a continuous key press. **(b)** Histograms of phase distributions for the dominant (DE, green) and non-dominant (NDE, blue) eye were pooled across participants. Data were normalised by the median dominant eye phase duration. **(c)** Average phase durations in seconds for DE (green) and NDE (blue) are plotted on the left. On the right are the biases in phase duration quantified for each participant by calculating the difference between the dominant and non-dominant eye phase duration as a percentage of the non-dominant eye phase duration. This index is henceforth referred to as ‘Eye Dominance [%]’ (clear bar, EDI).

For each participant, sensory eye dominance was measured individually using binocular rivalry stimuli presented by means of a Wheatstone stereoscope (Fig. 1a). Binocular rivalry phase distributions pooled across all observers were not normally distributed (Shapiro–Wilk Normality Test, W-stats = 0.64, *p* = 0.0001) as demonstrated by the typical skewedness^27^ of bistable percept phase durations (Fig. 1b). The median phase durations in seconds were used as a measure of central tendency^28^. Median phase durations across 12 observers were normally distributed (W-stats = 0.95, *p* = 0.337), hence parametric statistics were used for significance testing with an uncorrected alpha of 0.05. After confirming that there was no overall difference in right vs left eye median phase durations (two-tailed paired samples *t*-test, *t*(11) = 0.228, *p* = 0.824), eye dominance was assigned based on the length of the median phase duration (Fig. 1c, left; 5 observers = left eye dominant, 7 observers = right eye dominant, left vs right median). Not surprisingly, median phase durations were greater for the DE compared to the NDE (two-tailed paired samples *t*-test, *t*(11) = 2.960, *p* = 0.013). To obtain a singular measure of the increase in eye dominance, an eye dominance index (Fig. 1c right; ‘EDI’, see “Methods” section, Eq. (1)) was calculated. The EDI measures the increase in duration of the DE phase as a percentage of the NDE phase (range 0.64–23.1%, mean: 7.9 ± 8.2%).

### Monocular visual stimulation protocol during combined fMRI-MRS acquisition

**Figure 2:**
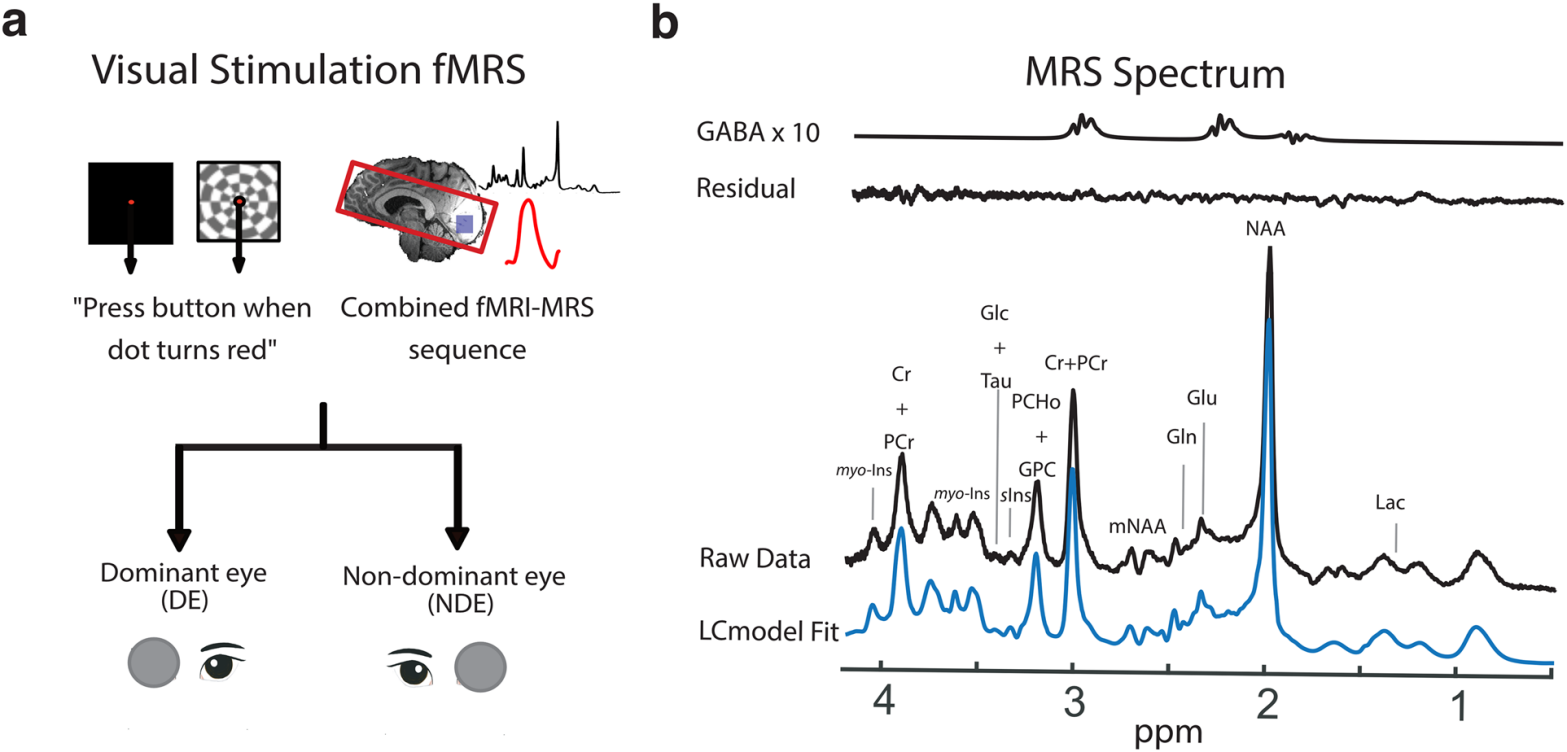
The combined fMRI-MRS monocular visual stimulation paradigm. **(a)** 7-Tesla combined fMRI-MRS data were collected from the early visual cortex during viewing of visual stimuli through the dominant or non-dominant eye. Visual input comprised of a constant fixation task, while a 50% contrast flashing checkerboard was cycled on (‘checkerboard’) and off (‘fixation’) in the background. **(b)** A representative MRS spectrum during viewing of visual stimuli through one eye, created by averaging across checkerboard and fixation periods. From bottom to top: blue spectrum shows LCModel fit to raw data. Black spectrum shows raw data. Immediately above are the residuals of the LCModel fit. Topmost line shows the LCModel fit to the GABA signal. For illustrative purposes, the GABA signal was multiplied by 10.

To measure cortical inhibition during monocular visual stimulation in the visual cortex (Fig. 2a), a 2 × 2 × 2 cm^3^ MRS volume-of-interest (VOI) was symmetrically placed along the midline of the calcarine sulcus to cover the early visual cortex (EVC) using a combined fMRI-MRS sequence (see ‘Supplementary Information’). We obtained high quality MRS spectra from the EVC volume (Fig. 2b), including detection of inhibitory neurotransmitter GABA**.** Across participants, an average of 85 ± 13.7% of the MRS VOI overlapped with the fMRI activation map (checkerboard > fixation), confirming that measurement of the neurochemical levels was measured from visually-stimulated tissue. No difference in percentage overlap was found between monocular stimulation conditions (dominant eye: 85 ± 13.7%, non-dominant eye: 86 ± 11.3%, Wilcoxon Rank Sum Test, *p* = 0.72).

### Interocular GABA from early visual cortex correlates with eye dominance

**Figure 3:**
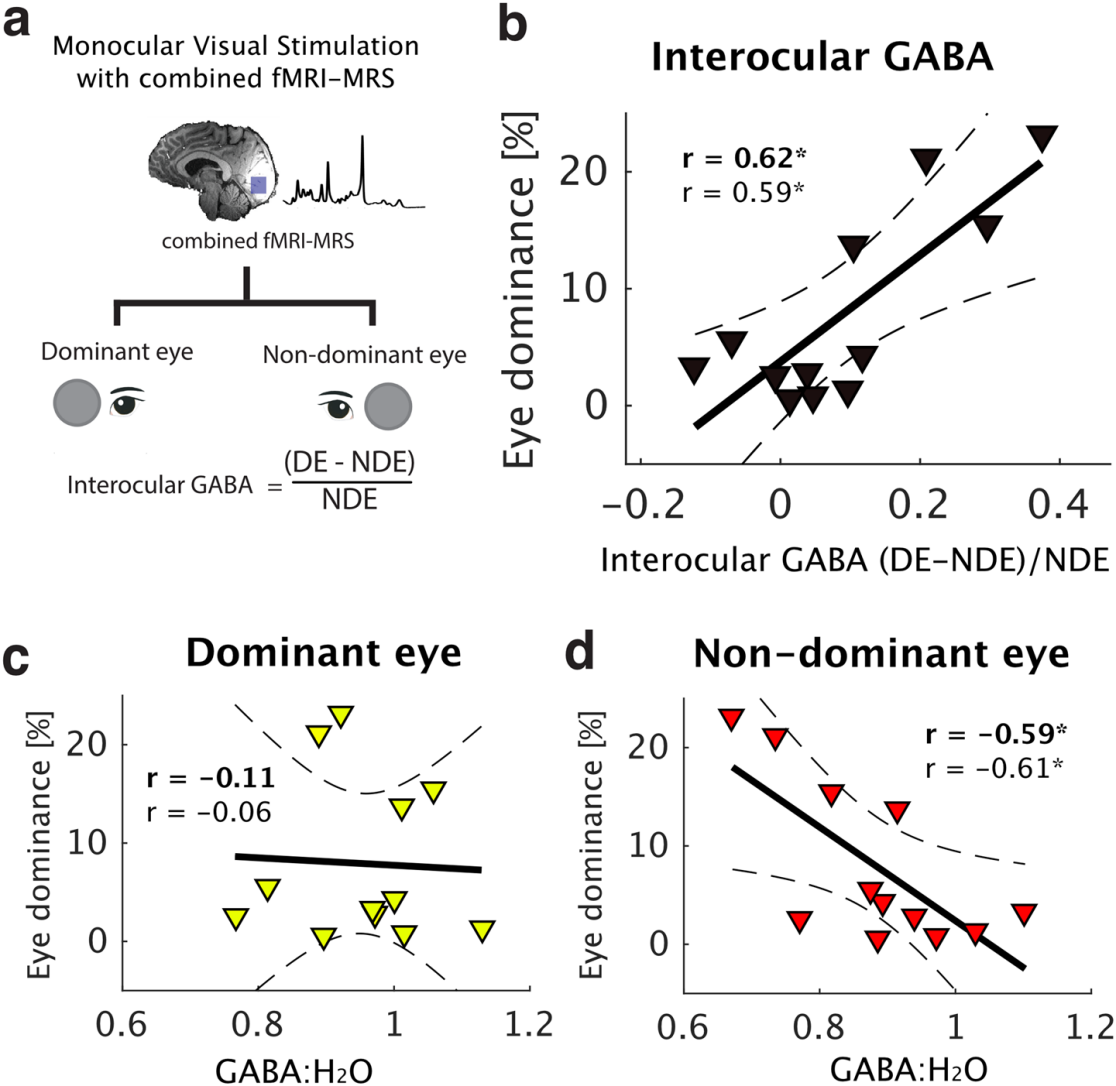
**(a)** The interocular GABA metric was calculated to show the difference in GABAergic inhibition between activation of the DE or NDE eye. **(b)** Eye dominance was highly correlated with interocular GABA. Further exploratory analysis showed that **(c)** eye dominance was not correlated with DE GABA:H_2_O, but that it was correlated with NDE GABA:H_2_O **(d)**. Solid line represents best fitting linear model, hatched lines represent 95% confidence interval of the regression line. Bold typed **r** = Spearman’s Rank Correlation Coefficient for GABA scaled to water, ‘*r*’ Spearman’s Rank Correlation Coefficient for GABA scaled to total Creatine, *uncorrected *p* < 0.05.

Mutual inhibition between neural inputs from each eye to the primary visual cortex is thought to drive eye dominance. To reveal differences in inhibitory responses between the dominant (DE) and non-dominant eye (NDE), participants alternately viewed visual stimuli with either their DE or NDE while the non-stimulated eye was covered with a semi-transparent occluder. Across participants, dominant eye GABA tended to be higher compared to NDE viewing, however the difference was not statistically significant (GABA:H_2_O, paired *t*-test, *t*(11) = 2.13, *p* = 0.06; GABA:tCr, paired *t*-test, *t*(11) = 1.72, *p* = 0.11). The difference in GABA levels between eyes was then quantified as a single metric, the ‘interocular’ GABA (Fig. 3a). We related the behaviourally derived eye dominance index (EDI), measured in the binocular rivalry experiment, to interocular GABA. EDI was significantly correlated with interocular GABA when using GABA scaled to the internal reference signal of unsuppressed water (Fig. 3b, GABA:H_2_O, Spearman’s Rank Correlation *r *= 0.62, uncorrected *p* = 0.037). To control for the possibility that the metabolite reference method influenced the results, we also scaled GABA to the summed signal of creatine and phosphocreatine (‘GABA:tCr’). The correlation remained significant (GABA:tCr, *r* = 0.59, uncorrected *p* = 0.049). In an exploratory analysis of this relationship, we show that there is no correlation between EDI and dominant eye GABA (Fig. 3c: Spearman’s Rank Correlation, GABA:H_2_O, *r* =  − 0.11, *p* = 0.73; GABA:tCr, *r* =  − 0.06, *p* = 0.83), but a negative association between EDI with non-dominant eye GABA (Fig. 3d: Spearman’s Rank Correlation, GABA:H_2_O, *r* =  − 0.59, *p* = 0.048; GABA:tCr, *r* =  − 0.61, *﻿p* = 0.037). We conclude that stronger eye dominance corresponds to a greater difference in GABAergic inhibition during dominant compared to non-dominant eye stimulation. Investigating the correlation within conditions suggested that the effect is partly accounted for by a failure of the NDE to inhibit the DE, as stronger eye dominance was associated with lower GABA during stimulation of the weaker eye.

Resting MRS data was also acquired while participants kept their eyes closed and no stimuli were delivered. We found no relationship between EDI and resting GABA (GABA:H_2_O, *r *=  − 0.14, *p* = 0.67; GABA:tCr, *r* =  − 0.09, *p* = 0.77). This control analysis shows that monocular visual stimulation was necessary to reveal inhibitory interactions relevant to eye dominance in the early visual cortex. Exploratory correlations between other metabolites and EDI are reported in the ‘Supplementary Information’ section in Table S1. We also tested whether the interocular difference in GABA levels (ΔGABA) was related to eye dominance. ΔGABA was calculated by comparing checkerboard > fixation prior to calculating the interocular ΔGABA metric. We found no correlation between eye dominance and interocular ΔGABA (*r* = 0.24, *p* = 0.44) suggesting that the addition of a checkerboard to fixation did not reveal a relationship to eye dominance.

### Eye dominance was not correlated with interocular BOLD-signal to flashing checkerboards

**Figure 4:**
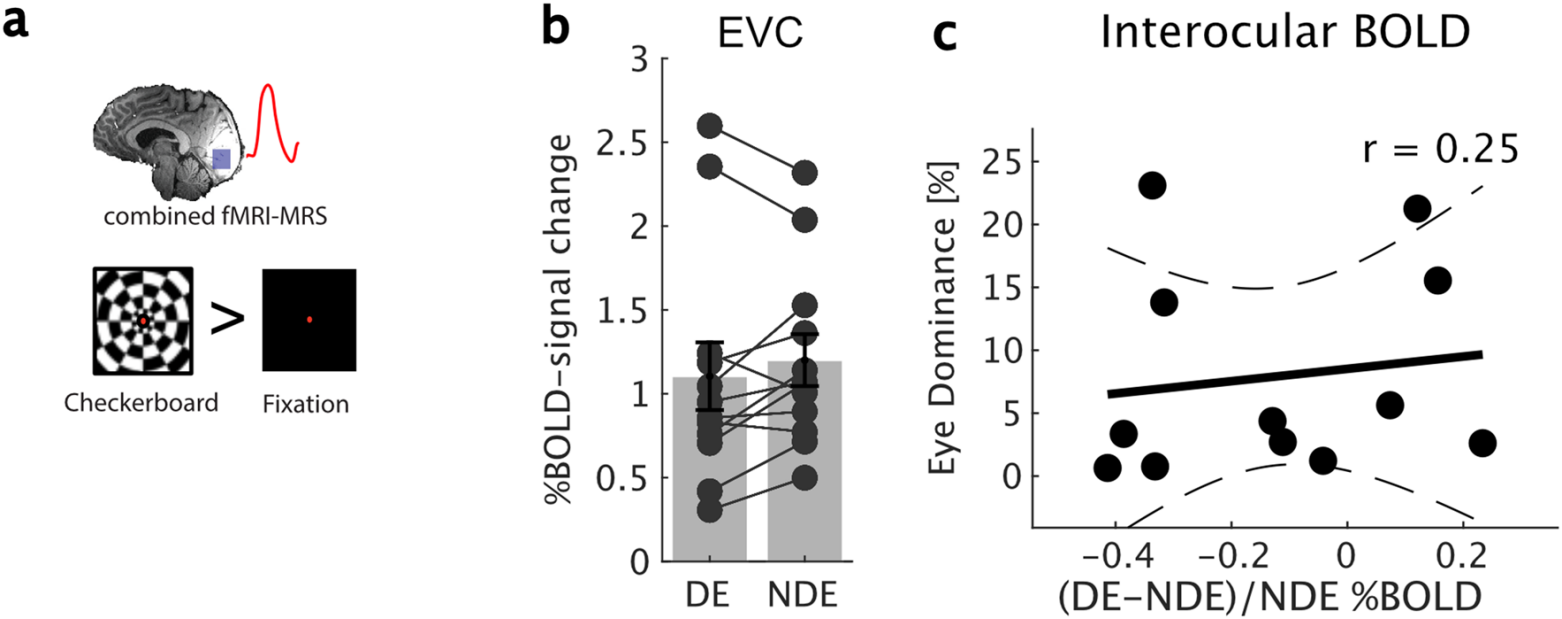
**(a)** %BOLD-change was obtained by comparing checkerboard > fixation prior to calculating the interocular BOLD metric. **(b)** %BOLD-signal change to a flashing checkerboard did not differ between dominant and non-dominant eye. **(c)** There was no relationship between eye dominance and the interocular %BOLD to the flashing checkerboard. Errors are 1 ± SEM. *r* Spearman’s Rank Correlation. Solid line represents best fitting linear model, hatched lines represent 95% confidence interval of the regression line.

The combined fMRI-MRS sequence measures fMRI and MRS data in the same TR. The next step was to investigate whether the BOLD-fMRI response from the same region in the early visual cortex also related to eye dominance. The complementary measures reflect different aspects of binocular function and require different analysis approaches. The fMRS analysis revealed how inhibitory signals differed between DE and NDE irrespective of checkerboard or fixation condition. On the other hand, the fMRI-signal reveals differences in BOLD signal (checkerboard > fixation) between DE and NDE (Fig. 4a). The fMRI depends on increased activation to a flashing checkerboard relative to fixation, before it is compared across DE and NDE eyes, and therefore cannot provide any information about activation to the fixation task alone. We found no difference in the checkerboard-evoked BOLD-signal between DE and NDE (Fig. 4b: Wilcoxon’s Rank Sum, *z* =  − 0.77; *p* = 0.43). We then calculated the interocular BOLD-signal (for ‘interocular BOLD’, see “Methods” section Eq. (3)). There was no correlation between EDI and interocular BOLD (Fig. 4c, Spearman’s *r* = 0.251, *p* = 0.430).

### Resting GABA relates to binocular rivalry suppression

**Figure 5:**
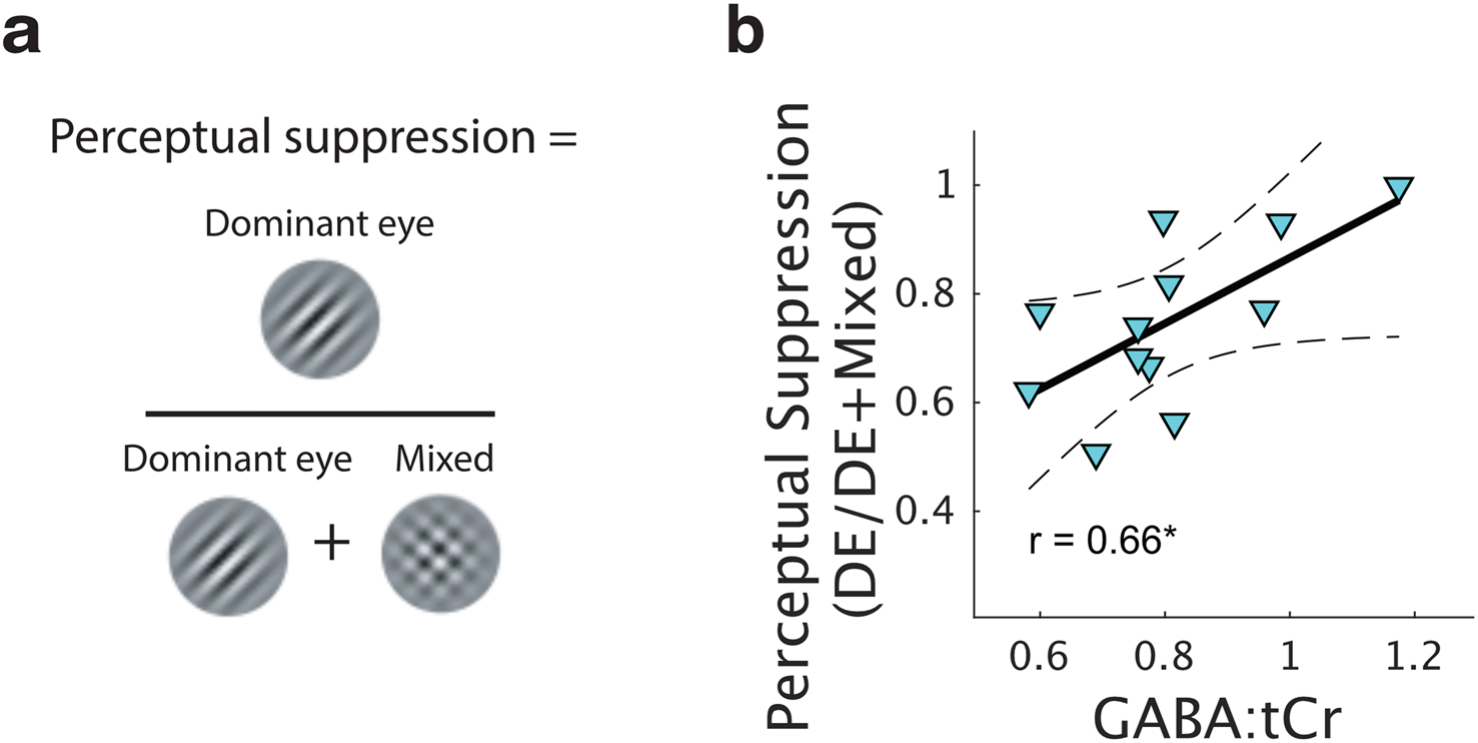
**﻿(a)** To compare with previous data, we calculated an alternative measure of binocular rivalry dynamics, the ‘perceptual suppression’ rate (Robertson et al.^17^). **(b)** Resting GABA:tCr levels was correlated with binocular rivalry perceptual suppression [dominant eye/(dominant eye + mixed percept)]. *r*= Pearson’s linear correlation co-efficient. *p < 0.05, uncorrected. Solid line represents best fitting linear model, hatched lines represent 95% confidence interval of the regression line.

Our dataset provided an opportunity to replicate a prior finding on binocular dynamics in the early visual cortex, obtained using different methods. Robertson et al., found a relationship between GABAergic inhibition and the proportion of perceptual suppression^17^. In their study, participants viewed stimuli with both eyes and performed a simple task at fixation, a state referred to as ‘resting MRS’. We used our resting GABA, when participants had their eyes closed and no stimulation was delivered, to attempt to replicate their findings. Using the same data from which we derived the eye dominance metric, we calculated binocular rivalry suppression, the proportion of the time seeing the dominant percept, divided by the sum of the dominant and mixed percept proportions (Fig. 5a). There was a significant correlation between perceptual suppression and resting GABA:tCr (Fig. 5b, *r *= 0.66, *p* = 0.02) although the correlation was not significant for GABA:H_2_O (*r* = 0.48, *p* = 0.12). This result suggests that the link between GABAergic inhibition and perceptual suppression can be replicated at ultra-high field strength, with the combined fMRI-MRS sequences and a different definition of ‘resting MRS’. The finding that GABA:tCr was correlated with binocular suppression while GABA:H_2_O was not, suggests that GABA levels may not be wholly independent from influences of Creatine energy metabolism^29^.

## Discussion

Eye dominance in the normal visual system may be regarded as a window into how the brain selects and combines information from the two eyes. Extreme eye dominance has a pathological form manifest in ‘amblyopia’, which impairs normal visual function. We demonstrate a link between intracortical inhibition and normal eye dominance in the healthy visual system, based on a novel approach to measurement of inhibition in the cortex. We applied monocular visual stimulation during combined fMRI-MRS at 7-Tesla. There was no link between eye dominance and inhibition in the simultaneously acquired BOLD-signal, or in resting GABA levels.

We show that GABA-levels measured during visual stimulation in the early visual cortex are linked to eye dominance in the healthy human brain. Our results are in agreement with the view that the neural mechanism of eye dominance is mediated by GABAergic inhibition^9,30^. With regard to the difference in GABA between DE and NDE eye (‘interocular GABA’), individuals with stronger eye dominance showed a greater difference in GABA. In an exploratory analysis, we further show that this difference is partially driven by a failure to inhibit the stronger eye during activation of the weaker eye. Eye dominance may therefore be due to a systematically weaker ability of the non-dominant eye to inhibit the stronger eye during active viewing. In confirmation, no relationship to eye dominance was present when GABA levels were measured during the resting state, during which participants had their eyes closed. Our control analyses (‘Supplementary Information’, Table S2) showed that spectral quality measures from our data (SNR, tCr line width), did not correlate with interocular GABA. In addition, interocular GABA related to eye dominance independently of the reference method. These results suggest that our main result was not driven by differences in spectral quality or the metabolite reference method.

What might the visually driven GABA signals represent? While the coarse spatial and temporal resolution of single-voxel MRS precludes a definite assignment of MRS-visible GABA to a particular cellular function or compartment, our functional paradigm associated GABA with neural activity during monocular visual stimulation. Monocular stimulation would have increased local metabolic demand and neurotransmitter release across neurons involved in visual processing and task-performance. Our analysis shows that the majority of the MRS VOI overlaps with functional activation and primary visual cortex (V1). While around 15–20% of the neurons in the cerebral cortex are inhibitory, a high percentage (~ 70–80%) of GABAergic neurons in V1 are parvalbumin expressing (PV +) interneurons^31,32^. The network of PV + interneurons in V1 has been extensively linked to early ocular dominance plasticity^33^. Thus, visually driven GABA signals observed in our study could represent intra-cortical inhibition from GABAergic interneurons that bias perception in favour of the dominant eye. Our findings here emphasize that revealing of inhibitory interactions in the binocular visual system depends on the status of visual input.

There was no relationship between eye dominance and the interocular BOLD-signal. It is important to point out that the BOLD-signal was dependent the contrast between checkerboard and fixation, whereas the fMRS GABA measures were calculated across both stimulus conditions. Hence, the two measures cannot be directly compared. Only a handful of studies have investigated the relationship between eye dominance and visual cortex BOLD-signal change and it is fair to say that the results have so far been inconclusive, partly due to differences in how eye dominance was assigned. Greater BOLD-activation in V1 has been found during DE versus NDE stimulation^34,35^, whereas others find no difference^36^. In these studies, eye dominance was assigned using other approaches, such as ‘sighting dominance’. Different methods of assigning eye dominance often do not agree with one another (for a review see^37^). We measured sighting dominance for all our subjects and found that it corresponded with assignments of sensory eye dominance assignments in only 7 out of 12 participants. A better comparison with our study may be found from a recent study using binocular rivalry: here they reported that increases in eye dominance induced by monocular deprivation are linked to increases in deprived eye BOLD-activation^38^. The lack of any correlation in our BOLD-signal data is consistent with the view that the BOLD-signal is not sensitive to interocular inhibition in early visual cortex^39^, whereas MRS has significant sensitivity to this signal^17,18,21^.

While we measured eye dominance using binocular rivalry in participants with normal stereo vision, a significant minority of the general population do not have normal binocular functions. Using a meta-analysis, Chopin et al.^40^ estimated that ~ 7% of the general population fall into this category, which can include those with binocular vision pathologies. More specifically, stereo-anomalous can be defined as individuals who fail stereo-vision tests, but unlike amblyopes, they do not exhibit a difference in visual acuity between eyes. In such individuals, mean binocular rivalry eye dominance durations were shown to be twice as long as those of stereo-normals^41^. It is well established that amblyopes have abnormal eye dominance^42^ that can approximate normal temporal patterns when vision in the fellow eye is attenuated using a neutral density filter^43^. More recently, sensory eye dominance has been used as an index for enhancement of binocular vision in amblyopes, with eye dominance modulated by visual perceptual training regimes^44,45^. Our result showing that eye dominance in the normal observer is correlated with GABAergic signalling could provide a basis for understanding the vision of individuals with greater asymmetry between eyes such as stereo-anomalous individuals and amblyopes.

Our study specifically set out to evaluate differences in interocular inhibition. However, it included a relatively small cohort (N = 12) and statistical results were not corrected for multiple comparisons. Nevertheless, our methods successfully replicated a recent result linking an alternate measure of interocular suppression and resting GABA levels, despite using a smaller sample size than the original study^17^. Additionally, our MRS-voxel size did not permit the spatial resolution to target the primary visual cortex alone. While a large percentage of the MRS voxel in early visual cortex co-localised to the primary visual cortex (47%), portions of V2 and V3 were also included. We therefore cannot rule out a contribution from visual areas beyond V1. Whilst we are confident that our protocol dissociated dominant from non-dominant eye stimulation at the input level, a long-term improvement in the spatial resolution of MRS imaging is required to resolve these signals at the circuit level in ocular dominance columns.

## Conclusion

In conclusion, these results have shown that GABAergic responses in the healthy human visual cortex relate to eye dominance. This relationship is specific to the conditions of visual stimulation and not present during GABA measured during rest. Although our study was conducted using a relatively small cohort of participants and should be interpreted with caution, we provide compelling evidence supporting a role of GABAergic inhibition in sensory eye dominance^9,15,16^. The extent to which these relationships can be related to clinical conditions of extreme eye dominance remains to be determined.

## Methods

### Participants

Thirteen participants (7 females, 29.2 ± 6.0 years, age range 21–42 years) including two of the authors took part in the study. All data presented were collected as part of a behavioural and MRI-data set, of which a subset has been published^25^. One participant was identified as an outlier due to a large percentage (68.5%) of mixed periods in the binocular rivalry experiment. Mixed periods are time points in which the participant reports seeing a piecemeal version of left and right eyes’ percept. Participants with a high percentage of mixed periods are excluded because they would not contribute enough data for analysis, may have an undiagnosed visual condition or an error in performing the task. Participants had normal or corrected-to-normal vision, no neurological impairments and normal stereo-acuity (< 120 arc s, TNO Stereo test, Lameris, Utrecht). All subjects were involved in a 1-h psychophysical session to measure binocular and monocular visual function, as well as eye dominance, and took part in a 1.5-h MRI session to measure interleaved changes in neurochemical levels and BOLD-activity. All subjects gave written informed consent. Approval for the study was obtained by the University of Oxford Research Ethics Committee (MSD-IDREC-C1-2014-146). The study was carried out in accordance to the Declaration of Helsinki.

### Behavioural protocol and procedure

Participants’ eye dominance was measured in a separate psychophysical session outside of the scanner (Fig. 1a). Sighting eye dominance was measured using the Miles Test^46^, which identifies the dominant eye as the one used for sighting of a distant target when viewed through an aperture. Sensory eye dominance was measured using binocular rivalry. While both sighting and sensory eye dominance tests were collected, only sensory eye dominance was used in subsequent analysis to provide a quantitative measure of eye dominance. Head position was stabilised with a chin and headrest. Stimuli were displayed on two gamma-linearised CRT monitors (viewing distance 57 cm) viewed through a Wheatstone mirror stereoscope. Stimuli were generated using MATLAB (The MathWorks, Natick, MA) with Psychophysics Toolbox^47^ running on an Apple Mac-mini. Binocular rivalry stimuli were two achromatic gratings (orientation: ± 45 deg off vertical, spatial frequency: 6 cycle/deg, contrast: 100%, diameter: 3.2 deg) presented on a uniform mid-grey background in the centre of vision. After successfully fusing a Nonius fusion target, participants self-initiated the trial and reported the perceived orientation of the tilted grating using the left (counter-clockwise) and right (clockwise) arrows of a computer keyboard; the upward arrow was pressed when a mixed percept was perceived. The orientations of the members of the grating pair were randomised across runs between the left and right eyes. After a practice run, all participants took part in three binocular rivalry runs, each lasting 180 s.

### Binocular rivalry analysis

Data were first pre-processed: missing data before perceptual transitions were assigned to the subsequent percept. Responses of < 200 ms, mixed responses or missing data with no response were removed from the analysis. Because of the skewedness of phase durations (Fig. 1b), the median phase durations were calculated^28^. To quantify sensory eye dominance, an ‘eye dominance index’ (EDI) was calculated as:

1 $${\text{EDI}} = \, \left[ {\left( {{\text{d}}_{{{\text{DE}}}} - {\text{ d}}_{{{\text{NDE}}}} } \right)/\left( {{\text{d}}_{{{\text{NDE}}}} } \right)} \right] \, \times { 1}00,$$

where d_DE_ is the median phase duration through the dominant eye and d_NDE_ is the median phase duration through the non-dominant eye. The EDI quantifies the percentage increase in median phase duration of the dominant over the non-dominant eye.

### Visual stimulation in MRI scanner

The visual stimulation paradigm continuously engaged neural pathways involved in visual processing (Fig. 3a). Identical visual stimulation was delivered to the dominant eye or the non-dominant eye. The non-viewing eye was occluded with a translucent, form-depriving occluder^21^. In order to minimise head motion, participants practised switching the occluder to the non-viewing eye prior to scanning and were encouraged not to give any verbal responses while in the scanner. To estimate GABA levels, data were analysed as a sustained visual stimulation design across the entire scan (128 spectral averages) during which a behavioural task at fixation was continuously present. Visual stimuli were comprised of the ‘checkerboard’ blocks, showing high contrast flashing checkerboards, and a ‘fixation’ block, showing an active fixation task presented on a black background. Participants performed an attention demanding task at fixation, during which they monitored the appearance of a red target and pressed a button on a button box as soon as possible. The task was performed continuously and irrespective of checkerboard or fixation blocks. Blocks with checkerboards present or absent were treated as the same for the MRS analysis, because the continuous fixation task alone is a form of visual stimulation that demands attention^48^ from monocular units. As a control analysis, we also performed the neurochemical analysis by subdividing the run into ‘checkerboard’ and ‘fixation’ blocks, and calculating the difference between block types.

Stimuli were generated on a Mac Book Pro laptop using Matlab and Psychtoolbox-3^47^ and displayed on a gamma-linearised Eiki LC-XL100 projector (resolution: 1024 × 768, refresh rate: 60 Hz). Participants viewed the stimuli through 45° angled mirrors on a back-projection screen (viewing distance: 60 cm). Visual stimuli were full-field checkerboards, contrast reversing at 8 Hz (stimulus size = 19.82° × 14.25°, 8 Hz flicker, mean luminance = 385 cd/m^2^; 50% contrast). The fixation condition was a uniform black screen (2.33 cd/m^2^) with a fixation dot task. Each run consisted of four alternations of fixation (64 s) followed by flashing checkerboards (64 s). A central fixation dot was displayed (white with black border, size = 0.75°) throughout the experiment. Participants were instructed to maintain fixation and press any button on a MRI-safe button box when the marker turned red (500 ms, ~ once in every 3 s).

### MR protocol

Magnetic Resonance data were acquired using a 7 T whole body (Siemens, Erlangen) MR-scanner with a Nova Medical head coil (single transmit, 32 receive channels). A T1-weighted structural scan was acquired for each participant with a 1-mm isotropic resolution (MPRAGE, repetition time TR = 2.2 s, inversion time T_I_ = 1.05 s, echo time TE = 2.82 ms, FOV = 192 × 192 × 176 mm, flip angle = 7°, total acquisition time = 171 s) to permit placement of the visual cortex voxel-of-interest (VOI). A 2 × 2 × 2 cm (8 cm^3^) MRS VOI was positioned to target the early visual cortex (EVC) in the occipital lobe. The EVC VOI was centred on the calcarine sulcus and at the midline to include V1 in both hemispheres. fMRI and MRS data were acquired using a combined fMRI-MRS sequence^25^, yielding a slab of EPI (3D EPI, resolution = 4.3 × 4.3 × 4.3 mm; flip angle = 5°, repetition time TR_epi_ = 40 ms, TE = 25 ms, FOV = 240 mm, 16 slices) and a MR spectrum upon each TR. The measurements used a single scan approach and were not phase-cycled, although there was a phase cycling step to minimize potential unwanted signals from outside of the VOI^49^. For each experimental run, 128 spectral averages were acquired using short-echo semi-localisation by adiabatic selective refocusing (semi-LASER) pulse sequence (TE = 36 ms, TR_mrs_ = 4 s) with VAPOR water suppression and outer volume suppression^50,51^. Data were collected as single free induction decay per excitation. A dielectric pad measuring 110 × 110 × 5 mm^3^ containing a suspension of Barium Titanate (BaTiO_3)_ and deuterated water (mass-mass ratio of 3:1) was placed behind the occiput of each participant^52,53^ to increase the effective transmit field efficiency close to the pad^54^.

### fMRI analysis

fMRI data were analysed using FEAT (FMRI Expert Analysis Tool) v.6.00, part of the FSL software distribution (FMRIB’s Software Library, www.fmrib.ox.ac.uk/fsl; RRID:SCR_002823). Pre-processing included motion correction MCFLIRT^55^; non-brain tissue extraction^56^; 5 mm smoothing, grand-mean intensity normalization and high pass temporal filtering (Gaussian-weighted least squares straight line fitting, main experiment = 132 s). Registration of EPI to an initial 2-mm structural image used 6 DOF, followed by registration to the 1-mm isotropic T1-weighted structural image using boundary-based registration (BBR) in FLIRT^55,57^. BOLD-change in the MRS-voxel was calculated using Featquery.

### fMRS analysis

Pre-processing for raw MRS data was performed in MRspa (https://www.cmrr.umn.edu/downloads/mrspa/) and included eddy current correction, frequency alignment to the tNAA singlet at 2.01 ppm and phase correction using a least-square algorithm prior to signal averaging. For each participant, 128 spectral averages were collected for each experimental condition. Metabolites within the chemical shift range of 0.5 to 4.5 were analysed using LCModel^58,59^. Group averages were calculated by averaging across LCModel metabolite estimates from each individual. Metabolite levels were estimated using a basis set of alanine (Ala); ascorbate/vitamin C (Asc); aspartate (Asp); glycerophosphorylcholine (GPC); phosphorylcholine (PCho); creatine (Cr); phosphocreatine (PCr); γ-aminobutyricacid (GABA); glucose (Glc); glutamine (Gln); glutamate (Glu); glutathione (GSH); inositol (Ins); lactate (Lac); phosphoeth anolamine (PE); scyllo-inositol (sIns); taurine (Tau); N-acetyl-aspartate multiplet (mNAA); N-acetyl-aspartate singlet (sNAA); Acetyl moiety of N-acetylaspartylglutamate (sNAAG); aspartyl moiety of NAAG (mNAAG); glutamate moiety of NAAG (gNAAG). Consistent with our previous studies^25,60^, the basis set allowed splitting of singlets and multiplets of NAA and NAAG spectra to account for differences in transverse relaxation and J-modulation at moderate echo times^61,62^. Macromolecule inclusion procedures were performed as in Bednarik et al.^23^. Macromolecular (MM) spectra were added in the LCModel basis set. MM spectra were measured from the occipital cortex of 3 participants using an inversion recovery sequence (TR = 3 s, TE = 36 ms, inversion time TI = 0.685 s), and were included in the model spectra. The residual signal of the methylene resonance of tCr at 3.93 ppm was removed by post processing and high-frequency noise was suppressed using a Gaussian filter (σ = 0.05 s) before including the MM spectrum into the LCModel basis set. A correlation coefficient is calculated to quantify the independence of metabolite estimates from each other. Two metabolites that have an absolute correlation coefficient > 0.5 cannot be separated from each other. In these cases, the sum rather than the pair is reported. Correlation coefficients across all metabolites were determined from the LCModel fitting of semi-LASER spectra. For example, the LCModel correlation coefficients were more negative than − 0.5 for the following pair of metabolites: Cr and PCr (r =  − 0.83).

The amount of cerebro-spinal fluid (CSF, 6.2 ± 2.3%), white matter (WM, 50 ± 3.9%) and grey matter (GM, 43.8 ± 2.9%) inside the EVC MRS voxel were estimated using the brain-extracted T1-weighted high resolution anatomy scan. Tissue fractions were determined by using FSL anatomical processing script fsl_anat and automated tissue segmentation (FSL v6.0 FAST^63^) with 5 mm Full-Width Half Maximum bias field correction. Percentage of tissue types were quantified by using the FSL command line tool fslstats. We identified the impact of BOLD-effects in metabolite spectra by estimating the total Creatine line width at 3.03 ppm (tCrLW) during dominant and non-dominant eye runs^64^. No differences in BOLD-effects between DE and NDE spectra were found (p = 0.82). Metabolites referenced to water were corrected for the amount of CSF using the equation: [M_corr]_ = [M] × (1/[1 − f_CSF_]), where M_corr_ is the corrected metabolite level, M the metabolite value from LCModel and f_CSF_ the CSF fraction^21,65^. GABA was scaled to the unsuppressed tissue water spectrum collected from the same MRS volume and at the start of each MRI session as a metabolite reference^66^. To reduce dependency of the GABA measure on tissue fraction, we applied the α-correction method^67–69^ by Harris et al.^67^, that first calculates GABA for a hypothetical all gray matter voxel, and then normalizes the values relative to the group tissue fraction using the equation:

2 $$c_{GMWMcorr} = \frac{{c_{{{\text{meas}}}} }}{{\left( {{\text{f}}_{{{\text{GM}}}} + \propto {\text{f}}_{{{\text{WM}}}} } \right)}}\frac{{\mu_{GM} + \alpha \mu_{WM} }}{{\mu_{GM} + \mu_{WM} }},$$

where c_GMWMcorr_ is the corrected value, c_meas_ is the uncorrected value, f_GM_ and f_WM_ are the participant’s GM and WM fractions, and α is the ratio of the metabolite estimate in grey and white matter, set to 0.5, and μ_GM_ and μ_WM_ are the GM and WM fractions across the group. An α-value of 0.5 has been chosen as white matter is assumed to have half the GABA level as gray matter^67^. Since the estimated GABA levels were not further corrected for tissue relaxation, we report metabolite levels scaled to unsuppressed water as ‘GABA:H_2_O’. As an alternative approach, GABA was also scaled to the sum of creatine and phosphocreatine (‘tCr’) signals acquired in the same voxel at the same time (‘GABA:tCr’)^70^. Referencing to tCr effectively controls for variations in tissue composition and CSF proportion across participants. GABA:tCr results are presented alongside results using GABA:H_2_O.

The quality of the MRS fits was quantified by the LCModel Cramér-Rao lower lounds (CRLB), and the criterion of 30% across participants (128 spectra/participant, N = 12) was chosen to exclude metabolites that were less detectable from noise. The averaged tissue corrected metabolite levels across 12 participants (128 spectra/participant) for GABA:H_2_O was 0.91 ± 0.12 (GABA:tCr, 0.81 ± 0.10, CRLB = 28.1 ± 3.7%). To assess the stability of GABA measurments over run time, we calculated the intra-subject test–retest reliability of GABA values using the coefficient of variation (CoV) (Supplementary Information, Fig S2). The intra-subject CoV was calculated by dividing the standard deviation of the measurements by their mean level^71^. GABA CoV was not affected by run time (Fig. S2b), supporting a stable measurement of GABA over acquisition time, nor was it affected by viewing condition (Fig. S2c).

The interocular difference metric in neural response is calculated for MRS measured GABA and for the %BOLD-signal change using the same equation:

3 $${\text{Interocular N }} = \, \left( {{\text{N}}_{{{\text{DE}}}} - {\text{N}}_{{{\text{NDE}}}} } \right)/{\text{N}}_{{{\text{NDE}}}} ,$$

where N_DE_ is the neural response value during dominant eye viewing, N_NDE_ is the neural response value during non-dominant eye viewing.

To quantify the percentage of the MRS VOI in respect to specific visual areas, we measured the percentage of the VOI that overlapped with cytoarchitectonic maps of human post-mortem data from bilateral V1, V2^72^, V3v and hV4^73^ using the FSL command line tool atlasquery (Supplementary Information, S1). No further thresholding was performed on the probabilistic atlases.

### Statistical analysis

Data were assessed for normality using the Shapiro–Wilk Test. If normality was rejected, (SW-test, *p* < 0.05) non-parametric statistics were used. The Wilcoxon Signed Rank test was used to test for significant differences in the median between two repeated measures. Spearman’s Rank correlation coefficients were calculated to evaluate the relationship between two independent variables of interest. The *p*-value was calculated using the exact distribution test. If normality was not refuted, paired *t*-tests were applied to test for differences between group means. Pearson’s Linear Correlation coefficients were computed to assess the relationship between two independent variables. In all cases, the significance value alpha was set to 0.05, uncorrected. Due to the low number of participants, it is possible that extreme data points influenced the data. However, no outliers were identified in metabolite or eye dominance measures using the function boxplot.m in Matlab.

## Supplementary Information

Supplementary Information.
