# Supplementary material for: GABAergic inhibition in the human visual cortex relates to eye dominance
Source: Sci Rep. 2021 Aug 23;11:17022. doi: 10.1038/s41598-021-95685-1 (PMC8382755; doi:10.1038/s41598-021-95685-1)
Supplement: Supplementary file 1 — Supplementary Information. [file 41598_2021_95685_MOESM1_ESM.docx]

**Supplementary Information**

**MRS VOI overlap with visual areas**


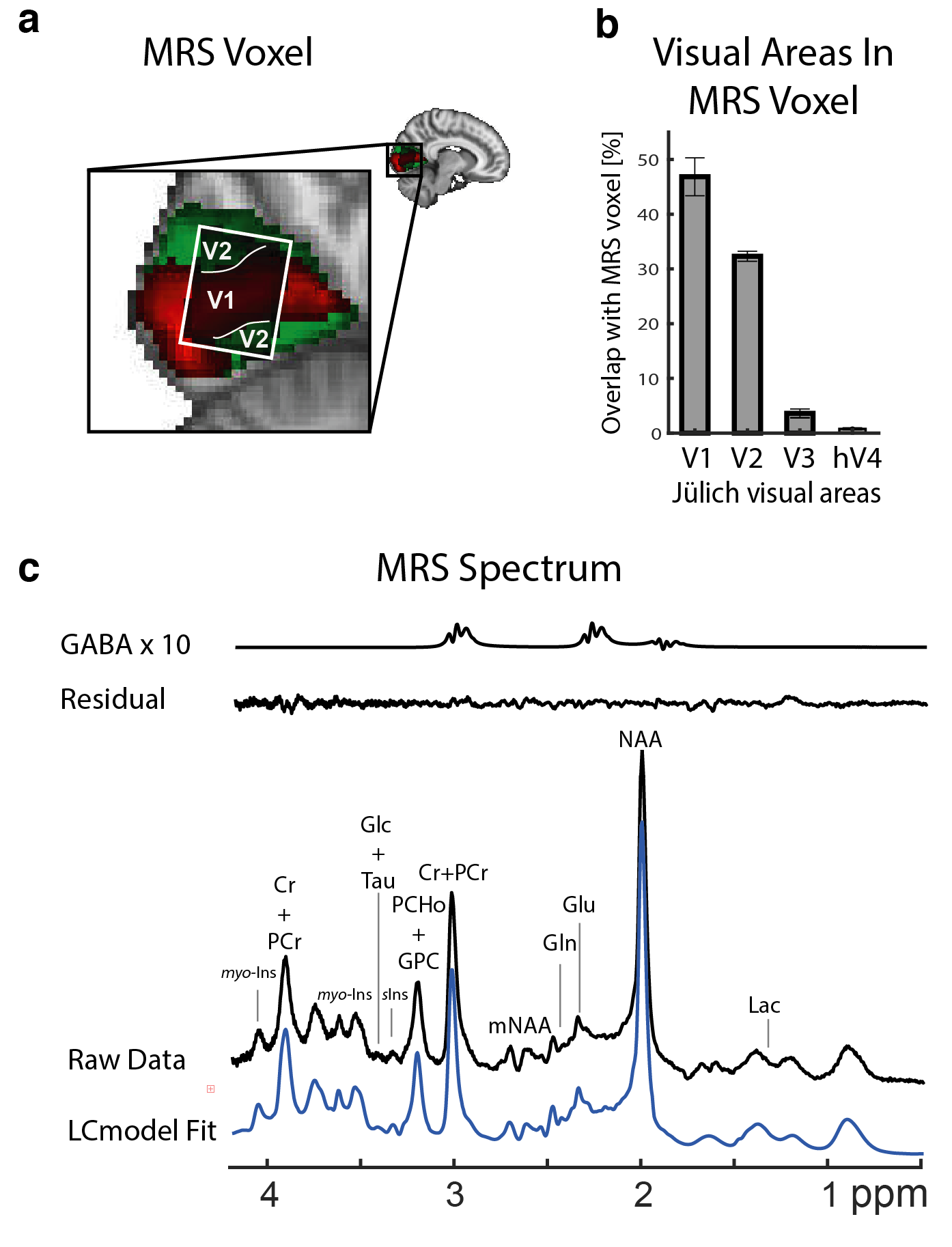
Fig S1a *Representative 2 x 2 x 2 cm MRS voxel (white box) overlaid onto V1 (red) and V2 (green) from the Jülich Atlas, displayed on the MNI-152 standard brain template. Atlas definitions were thresholded for display purposes. (b) Overlap between MRS voxel and visual areas (mean ± s.d.).*

To determine how much of the MRS VOI **(Fig.S1a)** overlapped with different areas in early visual cortex, we calculated the percentage overlap of the MRS VOI with V1-V3 and hV4 defined by the Jülich Atlas.

***Metabolite and eye dominance correlations***

In an exploratory analysis, we related other metabolites to our behavioural metric of eye dominance (EDI). The results are summarized in Table S1. A significant correlation, consistent across internal reference methods, was present only for GABA, scyllo-Inositol (sIns) and the combined signal of glucose and taurine (Glc+Tau). Glucose is a primary energy substrate for neuronal function, and increases in neuronal activity from the resting to an active state are accompanied by a rise in cellular energy production. 7-Tesla functional MRS studies have reported decreases in glucose levels during prolonged visual stimulation ^1-4^. These fluctuations are thought to reflect an increase in oxidative metabolism during neuronal activation ^2^. Our results are in agreement with previous functional MRS studies^1-4^, suggesting further that glucose metabolism is affected by the strength of eye dominance. sIns and Ins are markers of neuron-and astroglial function^5^ and can index astroglial contributions during sensory evoked neurotransmission and metabolism ^6^. Transient astrocytic volume changes can mark periods of intense functional activation^7^, and such changes have been demonstrated in the primary visual cortex in response to monocular stimulation^8^. Our result showing that sIns relates to eye dominance tentatively suggests that GABAergic signalling during competitive visual interactions could involve astrocyte metabolism in the visual cortex.  A more detailed investigation, taking advantage of specialized MRS imaging of astrocyte populations ^9^ and greater number of participants is needed to draw firmer conclusions.


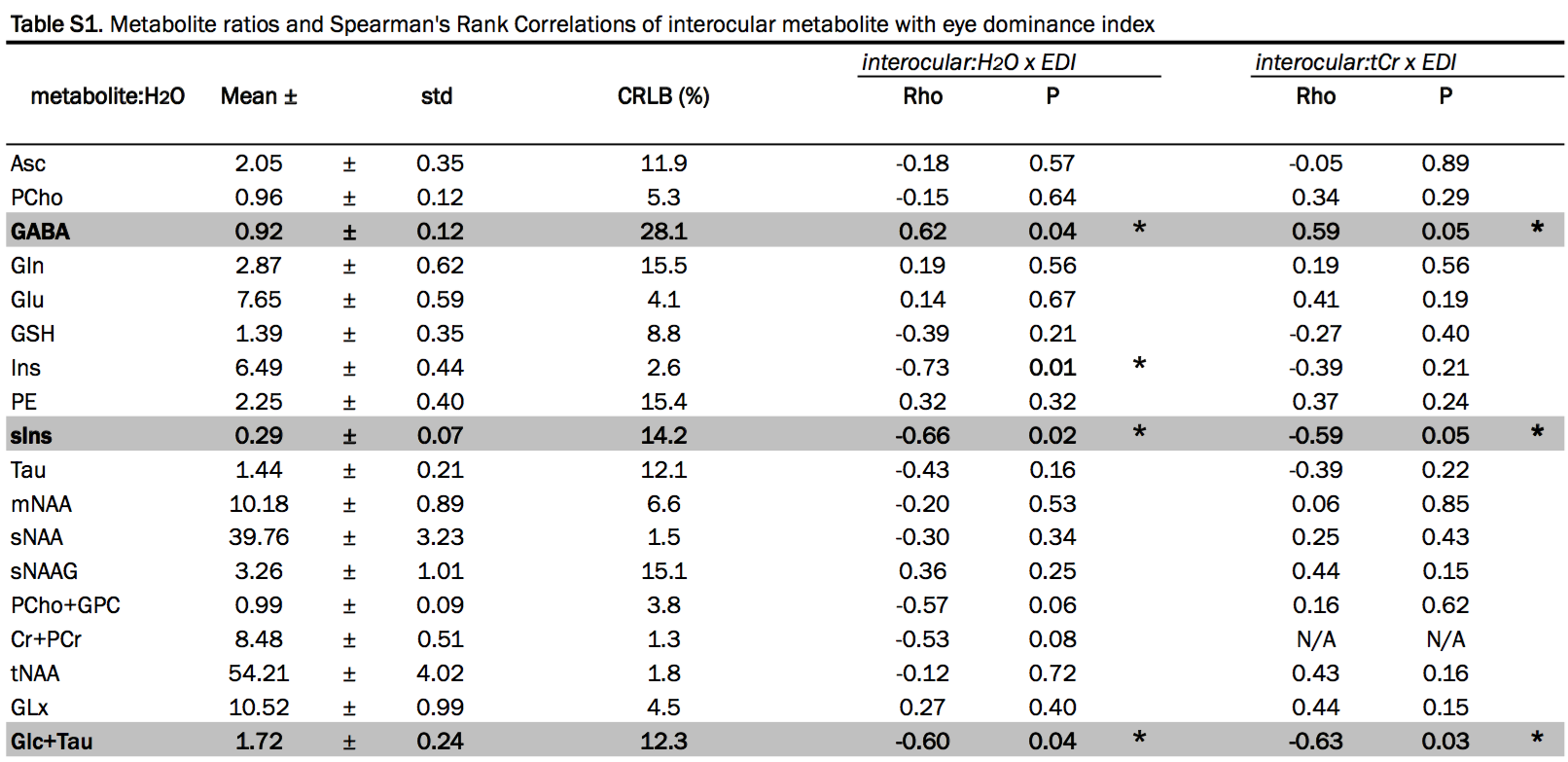


***Table S1.*** *First column reports average metabolite concentrations (mean ± std) scaled to internal water across dominant, non-dominant and resting MRS conditions. Second column reports the percentage Cramér-Rao Lower Bounds (CRLB%) as uncertainty estimates of LCModel fits (mean ± std). Third column presents Spearman’s Correlation Co-efficient (Rho) for interocular metabolite scaled to water (metabolite:H_2_O) with eye dominance index (EDI). Fourth column shows the same analysis, with metabolite concentrations scaled to the summed signals of creatine and phosphocreatine (tCr). P = p-value. * = uncorrected p ≤ 0.05.*

**Influence of spectral quality measures on GABA**

To evaluate the impact of spectral quality on GABA measures, we used two spectral quality measures: the signal-to-noise ratio (SNR), calculated as the height of the total N-Acetyl Aspartate (tNAA) peak divided by the standard deviation of a region of noise, and the line width of the total Creatine singlet at 3.03 ppm. We pooled values over non-dominant eye (NDE) and dominant eye (DE) conditions. The results are reported in Table S2. While we did not find a correlation between pooled SNR and pooled GABA, there was a correlation between pooled tCr LW and pooled GABA scaled to water (GABA:H_2_O, r = 0.41, p = 0.05). This correlation was dependent on the reference method, as there was no correlation between tCr LW and GABA:tCr (r = 0.21, p = 0.31). Prospectively, the influence of spectral quality on GABA:H_2_O could have accounted for the correlation between eye dominance and interocular GABA:H_2_O. To evaluate this possibility, we calculated the difference in spectral quality measures between eyes (‘interocular’ SNR and ‘interocular’ tCr LW) and correlated the measures with interocular GABA. Our results (Table 2), show that interocular spectral quality differences did not correlate with interocular GABA. Therefore, differences in spectral quality between eyes were unlikely to have driven the correlation between eye dominance and interocular GABA:H_2_O.


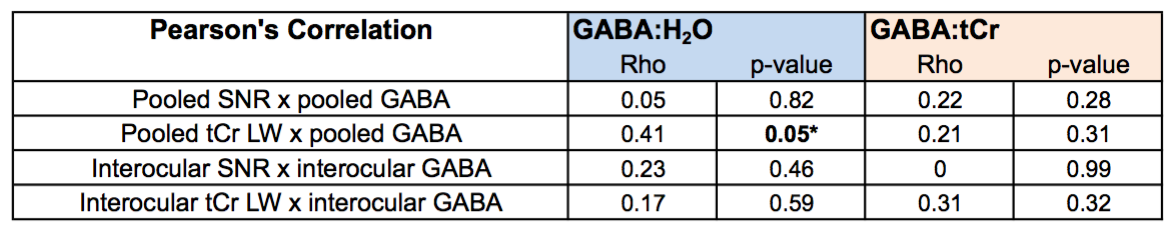


Table S2. Evaluating the relationship between spectral quality measures and GABA. SNR = Signal-to-noise ratio; GABA:H_2_O = GABA scaled to water; GABA:tCr = GABA scaled to the combined signal of creatine and phosphocreatine (‘total Creatine = tCr’); tCr LW = total Creatine singlet line width at 3.03 ppm; * = p < 0.05, uncorrected.

***Intra-subject GABA measurements are stable across scan duration***

Prospectively, the GABA measurements could have been affected by hardware instability and physiological noise during scan acquisition. To test how stable GABA measures were across scan duration and in different conditions, we quantified the percentage coefficient of variation (CoV)^1011^. The CoV was calculated by dividing the standard deviation of two measurements by their mean concentration. To obtain a CoV at regular intervals across scan duration, we split the data into four equal parts. The CoV was then calculated between the first and second part of the run and between the third and fourth part of the run (**Fig.** **S2a)**. We found no significant effect of scan duration on CoV (**Fig.** **S2b,** Wilcoxon Rank-Sum Test, p > 0.05), demonstrating that metabolite measurements were stable over the acquisition period. Next, we evaluated whether CoV differed by condition and found no difference (**Fig. S2c**, Friedman’s Test, p = 0.35), suggesting that GABA measurements were not affected by condition type. Average intra-subject CoV across conditions was 8.7 ± 2.9 % (mean ± std), in the lower range of previously reported within-subject CoV for GABA using non-edited MRS sequences [8-12%] ^12,13^. The relatively low intra-subject CoV suggests a greater ability to reliably detect GABA upon test-retest than estimated by the uncertainty of the GABA model fit ^11^. Our results are consistent with the increased accuracy of detecting low concentration metabolites such as GABA at ultra-high-field compared lower field strengths ^14^.


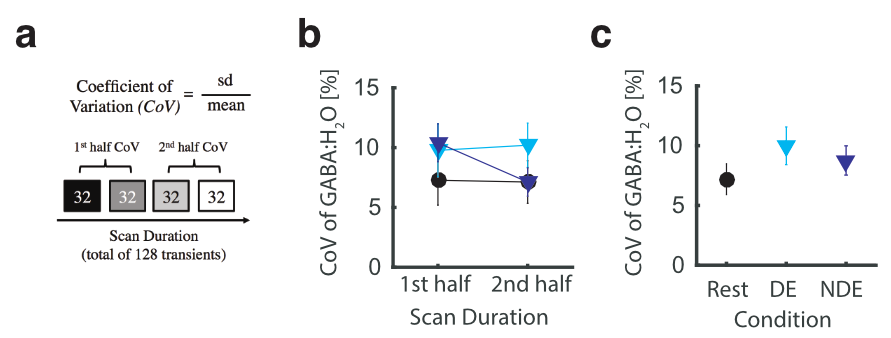


**Fig. S2:** Across scan duration reproducibility of GABA:H_2_O estimates. **(a)** CoV analysis was used to assess stability of GABA measures across scan duration. **(b)** GABA:H_2_O percentage Coefficient of Variation (CoV) for 1^st^ half and 2^nd^ half of the scan duration for each condition (black circle = resting GABA, cyan triangle = dominant eye, blue triangle = non-dominant eye)**. (c)** Average GABA:H_2_O CoV by condition. Data points show mean ± sem.

**References**

1 Schaller, B., Mekle, R., Xin, L. J., Kunz, N. & Gruetter, R. Net increase of lactate and glutamate concentration in activated human visual cortex detected with magnetic resonance spectroscopy at 7 tesla. *J Neurosci Res* **91**, 1076-1083, doi:10.1002/jnr.23194 (2013).

2 Mangia, S. *et al.* Sustained neuronal activation raises oxidative metabolism to a new steady-state level: evidence from H-1 NMR spectroscopy in the human visual cortex. *J Cerebr Blood F Met* **27**, 1055-1063, doi:10.1038/sj.jcbfm.9600401 (2007).

3 Bednarik, P. *et al.* Neurochemical and BOLD responses during neuronal activation measured in the human visual cortex at 7 Tesla. *Journal of cerebral blood flow and metabolism : official journal of the International Society of Cerebral Blood Flow and Metabolism* **35**, 601-610, doi:10.1038/jcbfm.2014.233 (2015).

4 Bednarik, P. *et al.* Neurochemical responses to chromatic and achromatic stimuli in the human visual cortex. *J Cerebr Blood F Met* **38**, 347-359, doi:10.1177/0271678x17695291 (2018).

5 Xu, H. Y. *et al.* Evaluation of neuron-glia integrity by in vivo proton magnetic resonance spectroscopy: Implications for psychiatric disorders. *Neurosci Biobehav R* **71**, 563-577, doi:10.1016/j.neubiorev.2016.09.027 (2016).

6 Perea, G., Yang, A., Boyden, E. S. & Sur, M. Optogenetic astrocyte activation modulates response selectivity of visual cortex neurons in vivo. *Nat Commun* **5**, https://doi.org/10.1038/ncomms4262

7 Macvicar, B. A. & Hochman, D. Imaging of Synaptically Evoked Intrinsic Optical Signals in Hippocampal Slices. *Journal of Neuroscience* **11**, 1458-1469 (1991).

8 Lu, H. D. D., Chen, G., Cai, J. J. & Roe, A. W. Intrinsic signal optical imaging of visual brain activity: Tracking of fast cortical dynamics. *NeuroImage* **148**, 160-168, doi:10.1016/j.neuroimage.2017.01.006 (2017).

9 Ligneu, C. *et al.* Diffusion-weighted magnetic resonance spectroscopy enables cell-specific monitoring of astrocyte reactivity in vivo. *NeuroImage* **191**, 457-469, doi:10.1016/j.neuroimage.2019.02.046 (2019).

10 Terpstra, M. *et al.* Test-retest reproducibility of neurochemical profiles with short-echo, single-voxel MR spectroscopy at 3T and 7T. *Magnetic resonance in medicine* **76**, 1083-1091, doi:10.1002/mrm.26022 (2016).

11 van de Bank, B. L. *et al.* Multi-center reproducibility of neurochemical profiles in the human brain at 7 T. *NMR in biomedicine* **28**, 306-316, doi:10.1002/nbm.3252 (2015).

12 Bogner, W. *et al.* In vivo quantification of intracerebral GABA by single-voxel (1)H-MRS-How reproducible are the results? *Eur J Radiol* **73**, 526-531, doi:10.1016/j.ejrad.2009.01.014 (2010).

13 Near, J. *et al.* Unedited in vivo detection and quantification of gamma-aminobutyric acid in the occipital cortex using short-TE MRS at 3 T. *NMR in biomedicine* **26**, 1353-1362, doi:10.1002/nbm.2960 (2013).

14 Tkac, I., Oz, G., Adriany, G., Ugurbil, K. & Gruetter, R. In Vivo H-1 NMR Spectroscopy of the Human Brain at High Magnetic Fields: Metabolite Quantification at 4T vs. 7T. *Magnetic resonance in medicine* **62**, 868-879, doi:10.1002/mrm.22086 (2009).
